# Antibacterial synergic effect of honey from two stingless bees: *Scaptotrigona bipunctata* Lepeletier, 1836, and *S. postica* Latreille, 1807

**DOI:** 10.1038/srep21641

**Published:** 2016-02-12

**Authors:** E. K. Nishio, J. M. Ribeiro, A. G. Oliveira, C. G. T. J. Andrade, E. A. Proni, R. K. T. Kobayashi, G. Nakazato

**Affiliations:** 1 Department of Microbiology, Center of Biological Sciences, Universidade Estadual de Londrina, Londrina, Paraná, Brazil, CP 6001; 2 Department of Animal and Vegetal Biology, Center of Biological Sciences, Universidade Estadual de Londrina, Londrina, Paraná, Brazil, CP 6001; 3 Department of General Biology, Center of Biological Sciences, Universidade Estadual de Londrina, Londrina, Paraná, Brazil, CP 6001

## Abstract

Several studies have tested antimicrobial activity of combinations of honey and various substances. In this study, we tested a combination of two stingless bee honeys against various bacterial strains. In particular: the antibacterial activity of honeys produced by *Scaptotrigona bipunctata* (SB) and *Scaptotrigona postica* (SP) was evaluated against Gram-positive and Gram-negative bacterial strains by agar well diffusion assays, minimum inhibitory concentration (MIC) assessment, construction of growth and viability curves and scanning electron microscopy (SEM). The interaction of the two honeys was also evaluated by the checkerboard assay. Inhibition zones ranged from 8 to 22 mm. The MIC values of the individual honeys ranged from 0.62 to 10% (v v^−1^) and decreased to 1/4 to 1/32 when the honeys were combined. SEM images showed division inhibition and cell wall disruption for the SB and SP honeys, respectively, and these alterations were observed in same field when the SB and SP honeys were combined. This study demonstrated that the natural honeys possess *in vitro* antimicrobial activity against Gram-positive and Gram-negative bacteria, including multidrug-resistant strains. Combination of the SB and SP honeys could lead to the development of new broad-spectrum antimicrobials that have the potential to prevent the emergence of resistant bacterial strains.

The emergence of multidrug-resistant bacteria and their rapid global spread are growing threats to public health^1^.The discovery of new antibiotics has decreased over the last few years, and multidrug-resistant bacteria have emerged during this period due to a negative correlation with the introduction of new anti-infectives^2^.

Honey has been used as a food since the earliest times, and it is considered to be part of traditional medicine. Indeed, apitherapy has gained attention as a form of folk and preventive medicine for treating diseases and promoting overall health^3^,^4^.

Despite the use of honey as an antibacterial in folk medicine, this practice has been replaced by synthetic and semisynthetic antimicrobials^5^,^6^. However, the indiscriminate use of antibiotics exerts a selective pressure on microorganisms, progressively selecting the most resistant^7^. Given this problem, the search for new antimicrobial compounds derived from different natural products, such as honey, to replace conventional antibiotic therapy is of high importance^8^,^9^,^10^.

Several studies have tested combinations of honey and various substances, including conventional antibiotics^11^,^12^,^13^, plant extracts^14^,^15^,^16^, royal jelly^17^ and propolis^18^,^19^. Although various combinations of honey and other compounds have been generated, the effect of the combination of different honeys on bacteria is still not known. In the present study, we tested a combination of two stingless bee honeys against various bacterial strains. These combinations could lead to the development of new broad-spectrum antimicrobials that have the potential to prevent the emergence of resistant bacterial strains.

## Results

### Agar well diffusion (AWD) of honey against Gram-positive and Gram-negative bacteria

As an initial screen to evaluate the antimicrobial activity of honey samples against bacterial strains, the AWD assay was applied, and inhibition zones were measured (Table 1). The inhibition zones of Gram-positive strains treated with *Scaptotrigona bipunctata* (SB) honey were 18.30 mm (standard error (SE) ± 1.07) on average, whereas Gram-negative strains exhibited 10.28 mm (SE ± 0.56) zones, indicating that Gram-positive bacteria were more sensitive (*p *< 0.05). A similar result was observed following treatment with *Scaptotrigona postica* (SP) honey: Gram-positive strains exhibited 13.90 mm (SE ± 1.17) zones on average, and Gram-negative bacteria yielded 8.14 mm (SE ± 0.24) zones (*p *< 0.05) (Table 1). However, when comparing the inhibition zones generated by SB honey combined with SP honey, no significant difference was observed between Gram-positive and Gram-negative bacteria (*p *> 0.05).

### Minimal inhibitory concentration (MIC) of honey

The average MIC values (Table 2) for Gram-positive bacteria treated with the SB and SP honeys were 1.87% (SE ± 0.39) and 2.50% (SE ± 0.81), respectively, whereas for Gram-negative bacteria, the values were 5.36% (SE ± 0.78) and 6.07% (SE ± 0.99) for the SB and SP honeys, respectively, with no significant differences between the honeys (*p *> 0.05) (Table 3). The MIC data corroborate that we observed in the AWD assay, which is that Gram-positive bacteria appear to be more sensitive to the antimicrobial effects than Gram-negative strains (*p *< 0.05).

### Combination of honeys

To determine the type of interaction that the SB and SP honeys present when combined, a checkerboard assay was performed. A synergistic antibacterial effect was observed against all bacterial strains when both the SB and the SP honeys were combined. The MIC values of the combination were reduced to 1/4 to 1/32 in relation to the MIC values of each honey alone (Table 3).

### Time-kill curve of *Staphylococcus aureus* against both honeys

**Figure 1:**
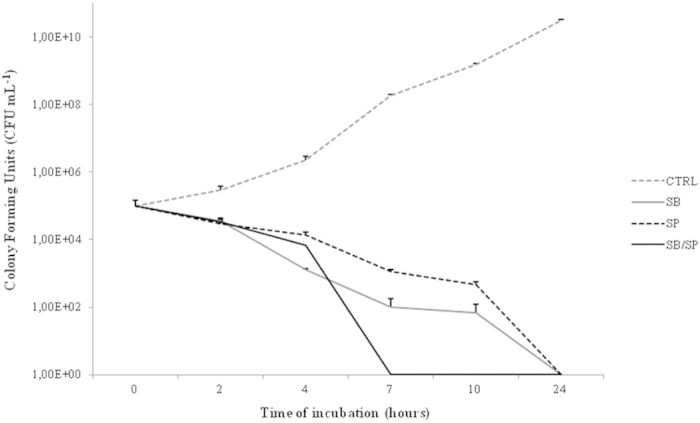
Time-kill curves of *S. aureus* strains exposed to honey at 1×MICs: SB honey used alone, 1.25%; SP honey used alone, 1.25%; and SB/SP honey used in combination, 0.31/0.31%. The control indicates bacterial growth without honey.

An evaluation of the kinetics of the honeys’ antibacterial effects against the methicillin-resistant *S. aureus* (MRSA) N315 strain was performed by constructing a time-kill curve. We observed a decrease of up to 4 log_10_ following 4 h of treatment compared with the control, but between treatments, there was no significant difference (*p *> 0.05) (Fig. 1). The treatment combining the honeys shortened the time needed for the elimination of all bacterial cells (Fig. 1).

### Non-peroxide activity

To evaluate non-peroxide activity, the honeys were treated with catalase to degrade any hydrogen peroxide. The average MIC values of the SB and SP honeys were respectively 13.00% (SE ± 1.73) and 14.00% (SE ± 1.85) for Gram-positive bacteria and 18.57% (SE ± 1.32) and 18.57% (SE ± 1.32) for Gram-negative bacteria (Table 2). Artificial honey showed no bacterial inhibition (MIC > 50%). When we compared the MIC values of honeys treated or not treated with catalase (*p *< 0.05), we observed a large contribution of hydrogen peroxide to the honeys’ antibacterial activity.

### Scanning electron microscopy (SEM) analysis of both honeys’ activity against *S. aureus*

**Figure 2:**
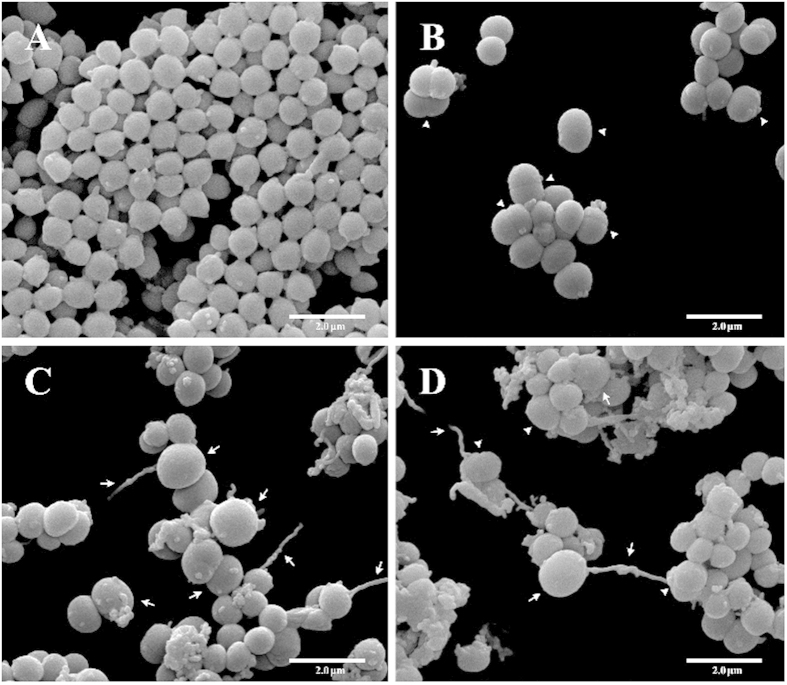
SEM images of the antibacterial effect of SB honey, SP honey and the combined SB/SP honeys against the MRSA N315 strain after 3 h of incubation. (**A**) negative control (without honey); (**B**) cells treated with SB honey (1.25%), showing that the cells formed septa (arrowhead); (**C**) cells treated with SP honey (1.25%), showing spheroplasts (arrow); (**D**) cells treated with both the SB (0.31%) and the SP (0.31%) honeys, showing that the cells formed septa (arrowhead) and spheroplasts (arrow). Different morphological alterations can be observed following treatment. The inset images show the detail of the morphological alterations.

An analysis of SEM micrographs of control cells (Fig. 2A) showed a large number of cells covering the entire field, with an intact structure, a uniform size (0.5 to 1.0 μm) and a large amount of extracellular matrix between the cells. After 3 h of incubation in the presence of honey, we observed a large reduction in the bacterial population for all treatments (Fig. 2B–D) compared with the control (Fig. 2A). Following treatment with SB honey, in 29% of cells, the presence of a septum was observed, but without cell division (Fig. 2B arrow head). Following treatment with SP honey, 16% of cells appeared to increase in size and exhibited cytoplasmic leakage (Fig. 2C arrow). Treatment with both honeys led to the occurrence of all of the events described above in 10% of cells in the field, which exhibited a cell division-related septum (Fig. 2D arrowhead), enlargement (Fig. 2D arrow) and cytoplasmic leakage.

## Discussion

Based on an AWD assay performed to evaluate the antibacterial activity of stingless bee honeys, Chan-Rodríguez *et al.*^20^ and Temaru *et al.*^21^ showed that Gram-positive bacteria may be more sensitive to these honeys compared with Gram-negative bacteria. Similar results were observed in our work for both the SB and the SP honeys, which were shown to be more effective against Gram-positive bacteria. Although the AWD assay is widely used to evaluate the antibacterial activity of honeys, several variables can influence the results^22^.

Garedew *et al.*^23^, who worked with honey from *Trigona* spp., and Boorn *et al.*^22^, who worked with honey from *Trigona carbonaria*, found MIC values ranging from 1 to 32% among Gram-positive bacteria and from 4 to 32% among Gram-negative bacteria. In our study, the MIC values were lower compared with those of the cited authors, with values ranging from 0.63 to 10% among Gram-positive bacteria and from 2.5 to 10% among Gram-negative bacteria. Determination of the MICs of different stingless bee honeys reaffirmed the data obtained from the AWD assay, showing that Gram-positive strains are more sensitive than Gram-negative bacteria are. Among the Gram-positive strains, *S. aureus* was associated with the lowest MIC values.

Different studies have evaluated the interaction of honey with other substances derived from bees, such as propolis^18^ and royal jelly^17^. In both of the cited studies, positive interactions were observed, but these can be considered as additive according to the fractional inhibitory concentration (FIC) index^24^. Our study showed that the combination of the SB and SP honeys involves a synergistic interaction, attaining an equal or even a better effect for a lower expense.

The kinetic evaluation of the antibacterial effect of stingless bee honeys from SB and SP showed a bactericidal effect. Similar results were observed by Temaru *et al.*^21^ using three stingless bee honeys (from *Melipona beecheii*, *Trigona biroi* and *Scaptotrigona pectoralis*). When the SB and SP honeys are used together, the time required to eliminate all bacteria is shorter than when the honeys are applied separately, showing a great advantage of this combination.

Several authors have described high osmolarity, acidity (low pH) and especially the hydrogen peroxide content as the main antimicrobial factors in honey^3^,^25^,^26^. In our study, artificial honey did not inhibit bacterial growth; the data specifically show that high osmolarity alone (simulated by artificial honey) is unable to inhibit bacterial growth.

Evaluation of the non-peroxide activity of honey by Kuncič *et al.*^27^ showed that when treated with catalase, Slovenian honey had 20-fold increased MIC values against Gram-positive and Gram-negative bacteria. In our study, when honey was treated with catalase, we observed a 5-fold increase in the MIC values. These data showed the importance of hydrogen peroxide to the antibacterial activity of the honeys used in this work, yet there are also other components present in these honeys that may inhibit bacterial growth.

*Melipona compressipes manaosensis* honey exhibits different antibacterial activity against Gram-positive and Gram-negative bacteria, depending on the season in which the honey is collected (wet or dry), with honey collected in the dry season having higher activity^28^. This difference was not observed in our study because the honeys used were collected throughout the year and because there was no significant variation in the antibacterial activity (data not shown).

Certain studies have analysed the physicochemical composition of honeys from SB and SP and have showed difference in their composition based on pH values (4.17 and 3.4), free acidity (34.63 and 83.7 meq kg^−1^), and hydroxymethylfurfural (HMF) content (2.5 and 18.9 mg kg^−1^)^29^,^30^. Studies have also indicated that the antibacterial activity varies according to the phytogeographic region, which leads to different compositions^31^,^32^.

Furthermore, new studies have indicated the presence of other antimicrobial components, such as methylglyoxal (MGO)^33^, antimicrobial peptide beedefensin-1^34^,HMF^35^and phenolic compounds such as flavonoids^36^.

Analysis of SEM micrographs allows us to observe morphological alterations caused by compounds placed in contact with bacteria; in our specific case, we examined the action of honey against *S. aureus*. When we observed images of cells treated with SB honey, we noted that a large number of cells formed septa, suggesting that effects on the cells make completion of the process of cell division unfeasible. Jenkins *et al.*^37^ exposed MRSA to Manuka honey and observed similar results by transmission electron microscopy (TEM). These authors suggested that honey may act on murein hydrolase by interfering with its post-translational modification, preventing hydrolysis of cell wall components^38^; therefore, a decrease in this enzyme’s activity would lead to failure in cell separation. Jenkins *et al.*^37^ further stated that synergy of MGO with other components present in honey may be responsible for effects on bacteria.

In the present study, bacterial cells treated with SP honey were shown to be larger than control cells. This cell enlargement may have occurred due to the partial disruption or degradation of the bacterial cell wall, leading to the formation of spheroplasts and therefore cell lysis and cytoplasmic leakage. Cushnie and Lamb^39^ tested the action of galangin (a flavonol present in honey) against *S. aureus* and found that the cells lost large amounts of potassium, similar to when treated with penicillin G. This phenomenon occurred due to inhibition of cell wall synthesis, reducing the mechanical strength of the cell wall. According to the authors, galangin may damage the cytoplasmic membrane directly or may weaken the cell wall, thereby causing osmotic lysis.

When we observe SEM micrographs of cells treated with both honeys, we noted alterations individually caused by the SB and SP honeys within the same field. Lorian and Fernandes^40^ tested a combination of two semisynthetic derivatives of pristinamycin, or quinupristin and dalfopristin, against *S. aureus*. These authors observed that when the derivatives were used individually, the bacterial cells appeared larger and were stained more intensely than the control; when used in combination, the cell size, the thickness of the cell wall, breaks in the wall and cell lysis increased. The honeys affected cells in different stages of the cell division cycle because peptidoglycan synthesis, an initial stage, was prevented (either completely or partially) by SP honey, preventing septum formation to separate dividing cells; thus, the honeys had a synergistic effect.

Although studies have increasingly sought to elucidate the active ingredients in honey, these ingredients are known they act synergistically. It may be that synergy of all ingredients brings about the maximum therapeutic efficacy, making honey as a whole a product of great interest.

There are few effective antimicrobials against multidrug-resistant bacteria, including MRSA strains. These antimicrobials are often associated with high costs and serious patient side effects. Several studies have combined honey with commercial antimicrobial drugs^12^,^13^, vegetable-origin natural products^41^ or animal-origin natural products^17^,^18^. Antibiotic combinations are advantageous and are commonly used for treatment. In many cases, these combinations are used to provide a broad spectrum of activity due to multitarget effects or to a delay in or suppression of the emergence of a drug-resistant population^42^,^43^. The current study demonstrated that *in vitro*, the combination of honeys possess antimicrobial activity against Gram-positive and Gram-negative bacteria, including multidrug-resistant strains; this combination has been patented^44^. Further studies are required to determine whether the combination has clinical applications for the treatment of infections.

## Materials and Methods

### Honey

The honey samples used in this study were collected from a university meliponary (Universidade Estadual de Londrina, Londrina-PR, Brazil) and Unidade de Conservação Monte Sinai (Mauá da Serra-PR, Brazil) during all seasons in 2013. The samples of honey called SB and SP were obtained from the stingless bees *Scaptotrigona bipunctata* (Lepeletier, 1836) and *Scaptotrigona postica* (Latreille, 1807), respectively. The honey samples were diluted in equal volumes of water (50% v v^−1^) and sterilized by filtration through a 0.22-μm filter (Millipore^®^).

### Bacterial strains

Reference strains of Gram-positive and Gram-negative bacteria were used, as follows: *Staphylococcus aureus* ATCC25923 and ATCC29213, *Staphylococcus epidermidis* ATCC12228, *Enterococcus faecalis* ATCC29212, *Enterococcus faecium* ATCC6569, *Streptococcus mutans* UA159, *Streptococcus pyogenes* ATCC19615, *Escherichia coli* ATCC25922 and ATCC8739, *Salmonella enterica* serovar Enteritidis ATCC13076, *Klebsiella pneumoniae* ATCC700603 and *Pseudomonas aeruginosa* ATCC27853 and ATCC9027.

Furthermore, the following other bacterial strains were used: methicillin-resistant *Staphylococcus aureus* (MRSA) N315 and BEC9393, which were provided by Dr. Elsa Masae Mamizuka (São Paulo University, São Paulo, São Paulo-SP, Brazil) and Dr. Agnes Marie Sá Figueiredo (Rio de Janeiro Federal University, Rio de Janeiro-RJ, Brazil), respectively, and *Salmonella enterica* serovar Typhimurium UK1, which was provided by Dr. Roy Curtiss III (Arizona State University, Tempe, AZ). All strains were stored at −80 °C in stocks containing glycerol (2.5 M).

### Agar well diffusion (AWD) assay

The AWD assay was performed in triplicate based on the work of Holder and Boyce^45^, with modification. Bacteria were first grown in Mueller-Hinton (MH) agar (Difco, USA), and incubated at 37 °C for 24 h. Each bacterial strain was suspended in sterile saline and adjusted to 0.5 on the McFarland scale, which corresponds to 1.5 × 10^8^ CFU ml^−1^. This suspension was spread on MH agar medium plates (thickness of approximately 4 mm). Following inoculation, a sterile glass borer was used to cut 6-mm wells into the surface of the agar. These wells were filled with 50 μl of honey 50% (v v^−1^). All plates were incubated at 37 °C for 24 h. After incubation, the diameters of the inhibition zones were measured.

### Minimum inhibitory concentration (MIC)

MICs were determined in triplicate by microdilution assays in 96-well plates according to the standards of the CLSI^46^. Bacterial suspensions were prepared in the same way described previously. These suspensions were diluted in MH broth (Difco^®^, USA) and plated in 96-well plates at a density of 5.0 × 10^5^ CFU well^−1^. Finally, different concentrations of honey solutions were added to each well to determine the MIC100 values. After the plates were incubated at 37 °C for 18 h, the optical density values were determined at 600 nm using a Bio-Rad Microplate Reader (model 3550).

### Checkerboard assay

To evaluate the interaction of SB honey and SP honey and to assess the antibacterial effects of the combination against bacterial strains, microdilution assays employing double antimicrobial gradient were used. The microdilution was performed in 96-well plates in triplicate according to the protocol of Kelly and Matsen^47^. To evaluate the interaction between the two antimicrobials, the fractional inhibitory concentration (FIC) index was used, as described by Chin *et al.*^24^.

### Time-kill assay

To evaluate the effect of honey samples on bacterial growth, a time-response growth curve was constructed according to the standards of the NCCLS^48^. First, a single colony-forming unit (CFU) of each methicillin-sensitive *S. aureus* (MSSA) or MRSA strain was inoculated in MH broth and grown for 18 h at 37 °C with constant stirring at 200 rpm. Second, each culture was adjusted to 0.5 on the McFarland scale and inoculated at a cell density of 10^6^ CFU ml^−1^ in two tubes, each containing 1 ml of MH broth. One culture received honey, and other culture did not receive honey (control). These cultures were incubated at 37 °C with constant stirring (200 rpm). Broth aliquots were collected at different time points, serially diluted in saline solution, plated on MH agar media and grown for 18 h at 37 °C to determine the total CFUs in each culture.

### Non-peroxide activity

To test non-peroxide antibacterial activity, catalase solution was prepared by dissolving 11000 units mg^−1^ solid catalase from bovine liver (Sigma) in distilled water, and this solution was added to the honey to a concentration of 2860 units ml^−1^ 21. The honey containing catalase was then used to determine the MIC value^46^. An artificial honey (75% w v^−1^), which was used as a control, was prepared by dissolving 32 g of fructose, 31 g of glucose, 12 g of maltose and 0.1 g of sucrose in 100 ml of distilled water, followed by sterilization at 121 °C for 15 min. This formulation reflects the approximate sugar composition of most stingless bee honeys^49^.

### Scanning electron microscopy (SEM)

SEM was used to observe the cell morphology. Colonies from the MRSA N315 culture grown on MH agar (24 h, 37 °C) were transferred to MH broth, and the cell density was adjusted to 10^8^ CFU ml^−1^. One millilitre of the cell suspension was distributed into 3 tubes, and honey (SB, SP or SB/SP) was added at the MIC. Culture in absence of the honey was considered as the control. The cultures were incubated at 37 °C and 150 rpm for 3 h. After incubation, 20 μl of each culture was collected and spotted onto polylysine-coated glass slides. Each slide was fixed by immersion in 1 ml of 2% glutaraldehyde and 2% paraformaldehyde in 0.1 M sodium cacodylate buffer (pH 7.2) solution for 20 h, followed by post-fixation in 1% OsO_4_ for 2 h. The fixed samples were dehydrated in an ethanol gradient (70, 80, 90 and 100 °CL) and then critical point dried in CO_2_ (BALTEC CPD 030 Critical Point Dryer). Finally, the slides were taped onto stubs, coated with gold (BALTEC SDC 050 Sputter Coater) and observed under an FEI Quanta 200 scanning electron microscope to analyse alterations in the cells.

### Statistical analysis

All data presented represent mean values from three replicate experiments. The data were analysed by one-way ANOVA, and differences among means were determined using the Qui-square test (α = 5%). All tests were performed with the statistical program BioEstat version 5.3.

## Additional Information

**How to cite this article**: Nishio, E. K. *et al.* Antibacterial synergic effect of honey from two stingless bees: *Scaptotrigona bipunctata* Lepeletier, 1836, and *S. postica* Latreille, 1807. *Sci. Rep.* **6**, 21641; doi: 10.1038/srep21641 (2016).

## Figures and Tables

**Table 1:** Diameters of the inhibition zones (mm) generated by SB and SP honey samples against Gram-positive and Gram-negative bacteria in an AWD assay.

Bacterial strain	Mean (±SE) inhibition zone size (mm)	
	SB	SP
*Enterococcus faecalis* ATCC29212	22 (±0.84)	18 (±0.34)
*E. faecium* ATCC6569	10 (±0.84)	7 (±0.34)
*Escherichia coli* ATCC25922	9 (±0.67)	8 (±0.31)
*E. coli* ATCC8739	8 (±0.67)	8 (±0.31)
*Klebsiella pneumoniae* ATCC700603	11 (±0.84)	7 (±0.34)
*Pseudomonas aeruginosa* ATCC27853	12 (±0.67)	8 (±0.31)
*P. aeruginosa* ATCC9027	11 (±0.67)	9 (±0.31)
*Salmonella enterica* Enteritidis ATCC13076	12 (±0.31)	9 (±0.63)
*S. enterica* Typhimurium UK1	9 (±0.31)	8 (±0.63)
*Staphylococcus aureus* ATCC25923	19 (±0.52)	15 (±0.30)
*S. aureus* ATCC29213	20 (±0.52)	16 (±0.30)
*S. aureus* methicillin-resistant BEC9393	20 (±0.45)	16 (±0.21)
*S. aureus* methicillin-resistant N315	19 (±0.45)	15 (±0.21)
*S. epidermidis* ATCC12228	20 (±0.45)	18 (±0.21)
*Streptococcus mutans* UA159	19 (±0.52)	11 (±0.30)
*S. pyogenes* ATCC19615	14 (±0.31)	8 (±0.63)

SE: Standard error.

ATCC: American Type Culture Collection.

SB: Honey from *S. bipunctata.*

SP: Honey from *S. postica.*

**Table 2:** MIC values of SB and SP honey samples used individually or in combination against Gram-positive and Gram-negative bacteria and the FIC values calculated to determine the type of honey-honey interaction.

Bacterial strain	MIC (%)			
	Without catalase		With catalase	
	SB	SP	SB	SP
*Enterococcus faecalis* ATCC29212	1.25	1.25	10.00	20.00
*E. faecium* ATCC6569	5.00	10.00	20.00	20.00
*Escherichia coli* ATCC25922	5.00	5.00	20.00	20.00
*E. coli* ATCC8739	5.00	5.00	20.00	20.00
*Klebsiella pneumoniae* ATCC700603	10.00	10.00	20.00	20.00
*Pseudomonas aeruginosa* ATCC27853	2.50	2.50	10.00	10.00
*P. aeruginosa* ATCC9027	5.00	5.00	20.00	20.00
*Salmonella enterica* Enteritidis ATCC13076	5.00	5.00	20.00	20.00
*S. enterica* Typhimurium UK1	5.00	10.00	20.00	20.00
*Staphylococcus aureus* ATCC25923	0.62	1.25	20.00	20.00
*S. aureus* ATCC29213	2.50	2.50	10.00	10.00
*S. aureus* methicillin-resistant BEC9393	0.62	1.25	10.00	10.00
*S. aureus* methicillin-resistant N315	1.25	1.25	10.00	10.00
*S. epidermidis* ATCC12228	2.50	2.50	10.00	10.00
*Streptococcus mutans* UA159	2.50	2.50	10.00	10.00
*S. pyogenes* ATCC19615	1.25	1.25	10.00	10.00

MIC: Minimum inhibitory concentration.

ATCC: American Type Culture Collection.

SB: Honey from *Scaptotrigona bipunctata.*

SP: Honey from *Scaptotrigona postica.*

**Table 3:** MIC values of SB and SP honey samples with or without catalase against Gram-positive and Gram-negative bacteria.

Bacterial strain	MIC alone (%)		MIC combined (%)		FIC	Interaction
	SB	SP	SB	SP		
*Enterococcus faecalis* ATCC29212	1.25	1.25	0.31	0.31	0.50	Synergic
*E. faecium* ATCC6569	5.00	10.00	0.63	2.50	0.38	Synergic
*Escherichia coli* ATCC25922	5.00	5.00	1.25	1.25	0.50	Synergic
*E. coli* ATCC8739	5.00	5.00	1.25	0.63	0.38	Synergic
*Klebsiella pneumoniae* ATCC700603	10.00	10.00	2.50	0.31	0.28	Synergic
*Pseudomonas aeruginosa* ATCC27853	2.50	2.50	0.63	0.63	0.50	Synergic
*P. aeruginosa* ATCC9027	5.00	5.00	1.25	0.63	0.38	Synergic
*Salmonella enterica* Enteritidis ATCC13076	5.00	5.00	1.25	1.25	0.50	Synergic
*S.enterica* Typhimurium UK1	5.00	10.00	1.25	0.63	0.31	Synergic
*Staphylococcus aureus* ATCC25923	0.62	1.25	0.16	0.31	0.50	Synergic
*S. aureus* ATCC29213	2.50	2.50	0.63	0.63	0.50	Synergic
*S. aureus* methicillin-resistantBEC9393	0.62	1.25	0.16	0.31	0.50	Synergic
*S. aureus* methicillin-resistantN315	1.25	1.25	0.31	0.16	0.38	Synergic
*S. epidermidis* ATCC12228	2.50	2.50	0.62	0.62	0.50	Synergic
*Streptococcus mutans* UA159	2.50	2.50	0.63	0.63	0.50	Synergic
*S. pyogenes* ATCC19615	1.25	1.25	0.31	0.31	0.50	Synergic

MIC: Minimum inhibitory concentration.

ATCC: American Type Culture Collection.

FIC: Fractional inhibitory concentration.

SB: Honey from *Scaptotrigona bipunctata.*

SP: Honey from *Scaptotrigona postica.*
